# Increased Risk of Depressive Disorder following Cholecystectomy for Gallstones

**DOI:** 10.1371/journal.pone.0129962

**Published:** 2015-06-08

**Authors:** Ming-Chieh Tsai, Chao-Hung Chen, Hsin-Chien Lee, Herng-Ching Lin, Cha-Ze Lee

**Affiliations:** 1 Division of Gastroenterology, Department of Internal Medicine, Cathay General Hospital, Taipei, Taiwan; 2 School of Health Care Administration, Taipei Medical University, Taipei, Taiwan; 3 Department of Cosmetic Applications and Management, Mackay Junior College of Medicine, Nursing, and Management, Taipei, Taiwan; 4 Department of Thoracic Surgery, MacKay Memorial Hospital, Taipei, Taiwan; 5 Sleep Research Center, Taipei Medical University Hospital, Taipei, Taiwan; 6 Department of Psychiatry, Taipei Medical University Hospital, Taipei, Taiwan; 7 Department of Internal Medicine, National Taiwan University Hospital and College of Medicine, Taipei, Taiwan; National Center of Neurology and Psychiatry, JAPAN

## Abstract

**Background:**

Prior studies indicate a possible association between depression and cholecystectomy, but no study has compared the risk of post-operative depressive disorders (DD) after cholecystectomy. This retrospective follow-up study aimed to examine the relationship between cholecystectomy and the risk of DD in patients with gallstones in a population-based database.

**Methods:**

Using ambulatory care data from the Longitudinal Health Insurance Database 2000, 6755 patients who received a first-time principal diagnosis of gallstones at the emergency room (ER) were identified. Among them, 1197 underwent cholecystectomy. Each patient was then individually followed-up for two years to identify those who were later diagnosed with DD. Cox proportional hazards regressions were performed to estimate the risk of developing DD between patients with gallstone who did and those who did not undergo cholecystectomy.

**Results:**

Of 6755 patients with gallstones, 173 (2.56%) were diagnosed with DD during the two-year follow-up. Among patients who did and those who did not undergo cholecystectomy, 3.51% and 2.36% later developed depressive disorder, respectively. After adjusting for the patient’s sex, age and geographic location, the hazard ratio (HR) of DD within two years of gallstone diagnosis was 1.43 (95% CI, 1.02–2.04) for patients who underwent cholecystectomy compared to those who did not. Females, but not males, had a higher the adjusted HR of DD (1.61; 95% CI, 1.08–2.41) for patients who underwent cholecystectomy compared to those who did not.

**Conclusions:**

There is an association between cholecystectomy and subsequent risk of DD among females, but not in males.

## Introduction

Gallstones, or cholelithiasis, are a common condition worldwide [1,2]. Its prevalence rate ranges from 10% to 15% in the United States and 3% to 10% for Asian countries [3,4]. Cholecystectomy is considered the curative intervention [5–7]. However, previous follow-up studies report that some residual gastrointestinal problems may continue to bother patients who had undergone cholecystectomy [5].

Studies reveal that after cholecystectomy, about 7–47% of patients are dissatisfied with the procedure, most of which are related to post-cholecystectomy syndrome (PCS) [8–13]. Previous studies report that PCS has a strong relationship with functional gastrointestinal disorders (FGIDs) [13–18]. Patients with FGIDs are reported to have a high prevalence of psychosocial disturbance [17–21]. Furthermore, persistent PCS have been correlated the feeling of depression [18,22–24]. Two small cohort studies report that the prevalence and incidence rates of depression following cholecystectomy are 2.7% and 3%, respectively [25,26]. A multi-center cohort study reports that 0.9% of patients have feelings of severe anxiety or depression and that 15% have mild depression one year after laparoscopic cholecystectomy [27]. One recent study also observes that 8% of patients with PCS caused by dysfunction of Oddi reportedly have depression an average of four years after cholecystectomy [16].

However, although prior studies indicate a possible association between depression and cholecystectomy, such studies have all relied on regional samples or on data from selected hospitals or sub-populations of patients. As such, these do not permit unequivocal conclusions. Furthermore, previous studies have focused on the relationship between cholecystectomy and the presentation of depressive symptoms, rather than on the diagnosis of depressive disorder (DD). To date, no study has attempted to compare the risk of post-operative depressive disorders following cholecystectomy. Therefore, this retrospective follow-up study aimed to examine the relationship between cholecystectomy and the subsequent risk of DD among patients with gallstone, using a population-based database.

## Materials and Methods

### Database

This retrospective cohort study utilized data retrieved from the Longitudinal Health Insurance Database (LHID2000). The LHID2000 was derived from the Taiwan National Health Insurance (NHI) program and included the medical claims for 1,000,000 enrollees randomly selected from all the enrollees (n = 23.72 million) listed in the 2000 Registry of Beneficiaries under the Taiwan National Health Insurance (NHI) program. Three domains were linked by the patient’s individual identity.

The LHID2000 consists of de-identified secondary data released to the public for research purposes by the National Health Research Institute. The LHID2000, which was open to the researchers in Taiwan, was available from the National Health Research Institute (http://nhird.nhri.org.tw/date_01.html). The LHID2000 has been utilized by numerous researchers, which have reported the high validity of data from the NHI program [28–30]. This study was approved by institutional review board (IRB) of Taipei Medical University's IRB (TMU-JIRB 201412008).

### Study Sample

There were 7213 patients who received a first-time principal diagnosis of gallstones (International Classification of Diseases, 9th edition, Clinical Modification (ICD-9-CM) code 574.0–574.4, 574.6–574.9) in emergency departments between January 1, 2001 and December 31, 2010. Among them, 1324 subsequently underwent cholecystectomy. The date of cholecystectomy was designated as the index date for patients who underwent a cholecystectomy. For those who did not undergo cholecystectomy (*n* = 5889), the date of the first gallstone diagnosis in the emergency department was defined as the index date. Patients who already had a diagnosed of DD (ICD-9-CM codes 296.2, 296.3, 300.4, and 311) prior to their index date were further excluded (*n* = 458). Ultimately, 6755 patients with gallstones were included.

Each patient was then individually followed-up for two years, beginning from the index date, to identify those who were diagnosed with DD within the follow-up period.

### Statistical Analysis

All statistical analyses were performed using the SAS statistical software (version 9.1 for Windows; SAS Institute, Inc., Cary, NC, USA). Statistical significance was set at *p*<0.05. The potential confounders were considered and included based upon both literature and statistical modeling. In previous studies on the issues of depression and cholecystectomy, these reported confounders may consist of characteristics of sex [31–34], age [31–34], and residential areas [32, 35]. Then, differences in sex, age group, monthly income (NT$ 0–15,840; NT$ 15,841–25,000, and NT$ >25,001), geographical location (Northern, Central, Eastern, and Southern Taiwan), and urbanization level of the patient’s residence (5 levels, 1 being the most urbanized and 5 being the least) between gallstone patients who did and those who did not undergo cholecystectomy were compared by Pearson Chi-square tests. Log-rank analysis was used to compare the difference in two-year DD-free survival rates between these two cohorts. We checked possible influential observations and assured that none deletion from the dataset would noticeably change the results presented.

In addition, Cox proportional hazards regressions were conducted to estimate the risk of developing DD during the two-year follow-up period between gallstone patients who underwent and those who did not undergo cholecystectomy, with cases censored if individuals were lost to follow-up during that time. Of all 334 censored cases during the follow-up period, 62 were from gallstone patients who underwent cholecystectomy and 272 were from gallstone patients who did not undergo cholecystectomy. Interaction terms were added to the Cox regression models to examine potential modifying effects of demographic characteristics. As the association between the risk of developing DD between gallstone patients who underwent and those who did not undergo cholecystectomy was different in males and in females, further analysis were performed stratified by sex. Hazard ratios (HR) and their corresponding 95% confidence intervals (95% CI) were used to report the risk of DD.

Finally, we checked the proportionality assumptions for Cox models. In the graphs with the survival function versus survival time, the shapes of the curves of the predictor (gallstone patients with and without undergoing cholecystectomy) were basically the same and the separation between the curves remained proportional over time. We further generated the time dependent covariates by creating interactions of the predictors and a function of survival time and included in the model. None of the time dependent covariates were significant. We examined for each covariate as well as globally to ensure the conformance with the proportionality assumption. In addition, the assumption of uninformative censoring was considered. We found no statistical significance in examining baseline characteristics of those who censored and retained.

## Results

The patients had a mean age of 52.0±16.9 years, 53.9±15.5 and 51.6±17.1 years for patients who underwent and those who did not undergo cholecystectomy, respectively (*p*<0.001, Table 1). There were no significant differences in monthly income (*p* = 0.180) and urbanization level of the patients’ residence (*p* = 0.351) between patients who underwent and those who did not undergo cholecystectomy. However, patients who underwent cholecystectomy had a significantly greater tendency to reside in northern part of Taiwan (*p*<0.001) than those who did not undergo cholecystectomy.

**Table 1:** Demographic characteristics for the sampled patients with gallstones, stratified by the presence/absence of cholecystectomy, 2001–2010 (n = 6,755).

Variable	Patients undergoing cholecystectomy N = 1,197		Patients did not undergo cholecystectomy N = 5,558		*P* value
	Total No.	Column %	Total No.	Column %	
**Male**	455	38.0	2,294	41.3	0.037
**Geographic region**					<0.001
** Northern**	540	45.1	2298	41.4	
** Central**	254	21.2	1572	28.3	
** Southern**	387	32.3	1613	29.0	
** Eastern**	16	1.3	75	1.3	
**Age group (years)**	53.9 ± 15.5		51.6 ± 17.1		<0.001
**Urbanization level**					0.351
** 1**	362	30.2	1712	30.8	
** 2**	269	30.8	1599	28.8	
** 3**	181	15.1	825	14.8	
** 4**	152	12.7	818	14.7	
** 5**	133	11.1	604	10.9	
**Monthly income**					0.180
** NT$ 0–15,840**	465	38.8	2294	41.3	
** NT$15,841–25,000**	456	38.1	1968	35.4	
** ≥NT$25,001**	276	23.1	1296	23.3	

In terms of the incidence of DD within the two-year period following the index date (Table 2), 173 out of 6755 (2.56%) gallstone patients were diagnosed with DD within the two-year follow-up period. In total, 3.51% and 2.36% of patients who did and did not undergo cholecystectomy subsequently diagnosis of DD, respectively. Log-rank test revealed a statistically significant difference in two-year DD-free accumulated survival rate between the two groups (Chi-square value = 5.216, *p* = 0.022).

**Table 2:** Crude and covariate-adjusted hazard ratios for depressive disorder among the sampled patients with gallstones during the two-year follow-up starting from the index date.

Presence of depressive disorder	Total sample N = 6755		Patients undergoing cholecystectomy N = 1197		Patients did not undergo cholecystectomy N = 5558	
	No.	%	No.	%	No.	%
**One-year follow-up period**						
** Yes**	173	2.56	42	3.51	131	2.36
** Crude HR (95% CI)**	-		1.51* (1.07–2.14)		1.00	
** Adjusted HR** ^**a**^ **(95% CI)**			1.43* (1.02–2.04)		1.00	

Note: HR, hazard ratio

^a^Adjustments are made for patient’s sex, age and geographical location

*p<0.05

The crude and adjusted HRs for DD during the two-year follow-up period were also shown in Table 2. Cox proportional analysis indicated that patients who underwent cholecystectomy had higher risk of developing DD compared to patients who did not undergo cholecystectomy (HR 1.51; 95% CI, 1.07–2.14). After adjusting for the patients’ geographical location and censoring those were lost to follow-up, the HR of DD within the two-year follow-up period was 1.43 (95% CI, 1.02–2.04) for patients who underwent cholecystectomy compared to those who did not.

The HR of the two groups as stratified according to sex revealed that for females, the adjusted HR of DD for patients who underwent cholecystectomy was 1.61 (95% CI = 1.08–2.41) compared to patients who did not undergo cholecystectomy (Table 3). There were no similar findings for males (adjusted HR, 0.95; 95% CI, 0.44–2.06).

**Table 3:** Crude and covariate-adjusted hazard ratios for depressive disorder among the sampled patients with gallstones in the two-year follow-up starting from the index date, stratified by sex.

Presence of depressive disorder	Male		Female	
	Patients undergoing cholecystectomy N = 455	Patients did not undergo cholecystectomy N = 2294	Patients undergoing cholecystectomy N = 742	Patients did not undergo cholecystectomy N = 3264
	n, %		n, %	
**Yes**	8 (1.76)	40 (1.74)	34 (4.58)	91 (2.79)
**Crude HR (95% CI)**	1.01 (0.47–2.17)	1.00	1.67* (1.12–2.50)	1.00
**Adjusted HR** ^**a**^ **(95% CI)**	0.95 (0.44–2.06)	1.00	1.61* (1.08–2.41)	1.00

Notes: HR, hazard ratio

^a^Adjustments were made for patient’s geographical location

*p<0.05

## Discussion

This retrospective cohort study shows that 3.51% of patients with gallstones who undergo cholecystectomy received a diagnosis of DD within two years from the diagnosis of gallstones. Among patients with gallstones, those who undergo cholecystectomy also have a significantly higher risk of DD than those who do not (adjusted HR, 1.43).

The current findings parallel those of prior studies, all of which reported that 0.9% to 3.0% of patients develop depression following cholecystectomy [25–27]. The high prevalence of depression among post-cholecystectomy patients may be explained by post-cholecystectomy syndrome (PCS), which may be a factor leading to depression. As the name implies, PCS involves abdominal symptoms that range from mild, ill-defined digestive symptoms to severe attacks of abdominal pain and jaundice that recur or persist after cholecystectomy [8,36–38]. According to different methods of evaluation and variable recorded symptoms, PCS occurs in up to 50% of patients, with 10–15% as the most reasonable range [8,12,37–39]. Reports also show that about 50% of PCS are affected by psychosomatic or extra-intestinal disease [39,40]. Previous studies report that PCS are significantly associated with a long history of complaints (e.g., biliary pain, symptoms, and attacks) [12,15,22]. Thus, PCS may be a possible explanation for the increasing risk of DD following cholecystectomy. The present study further analyzed the data but failed to establish a relationship between PCS and the subsequent risk of DD within the two-year follow-up period (adjusted HR = 0.71, 95% CI = 0.08–4.36).

Another mechanism may be the increased risk of metabolic syndrome and non-alcoholic fatty liver disease (NAFLD) after cholecystectomy [41,42]. Previous studies suggest that resistance to insulin effects on central neurons play a role in depression [43,44]. In addition, according to the DSM-IV criteria, DD is significantly related to insulin resistance as indexed by the homeostasis model assessment method [45]. The correlation between insulin resistance and depression has also been established by one intervention study, wherein insulin sensitivity is improved by treating depression [46]. Thus, cholecystectomy itself may be a risk factor and pathogenic link between insulin resistance and DD.

The present study has found an increasing risk of DD after cholecystectomy only in females, but not in males. One prior study suggests that female sex is significantly negatively associated with post-cholecystectomy health-related quality of life (HRQOL), as evaluated by the GIQLI and short Form-36 after a six-month follow-up [7]. Previous studies consistently report that PCS occurs frequently in women, while middle-aged women also have a high prevalence of DD [1,2,8].

This study has several strengths. First, a nationwide population-based dataset with ample sample size is used to clarify the relationship between cholecystectomy and the subsequent risk of DD. Second, DD based on established clinical diagnostic criteria is used rather than the presentation of depressive symptoms reported by survey. The diagnosis of DD has a very high validity since mental illness is still culturally taboo in Taiwan. A physician will not make a diagnosis of DD unless there is relative certainty.

Nonetheless, there are two limitations in the present study. First, there are no records of concomitant functional dyspepsia, which may contribute to the increased incidence of DD in post-cholecystectomy patients. Second, the LHID2000 does not provide information on operative time, gallbladder specimen histology, and the patient’s employment, marital status, educational level and body mass index (BMI). All of these may influence the outcome of cholecystectomy [6,7,37]. Since patients undergoing cholecystectomy are more likely to have frequent out-patient consults, which may lead to an early detection of DD, there may be a possible surveillance bias.

Despite the aforementioned limitations, this study demonstrates an association between cholecystectomy and subsequent risk of DD. Clinicians must be alert to the increased prevalence of clinical depressive symptoms in patients who underwent cholecystectomy.
